# Stress and Memory: Behavioral Effects and Neurobiological Mechanisms

**DOI:** 10.1155/2007/78970

**Published:** 2007-04-15

**Authors:** Carmen Sandi, M. Teresa Pinelo-Nava

**Affiliations:** ^1^Brain Mind Institute, Ecole Polytechnique Fédérale de Lausanne (EPFL), 1015 Lausanne, Switzerland; ^2^Departamento de Psicobiología, Universidad Nacional de Educación a Distancia, Juan del Rosal s/n, 28040 Madrid, Spain; ^3^Departamento de Psicología, Universidad Iberoamericana, Prolongación Paseo de la Reforma 880, Santa Fe, 01219 México, Mexico

## Abstract

Stress is a potent modulator of learning and memory processes. Although there have been a few attempts in the literature to explain the diversity of effects (including facilitating, impairing, and lack of effects) described for the impact of stress on memory function according to single classification criterion, they have proved insufficient to explain the whole complexity of effects. Here, we review the literature in the field of stress and memory interactions according to five selected classifying factors (source of stress, stressor duration, stressor intensity, stressor timing with regard to memory phase, and learning type) in an attempt to develop an integrative model to understand how stress affects memory function. Summarizing on those conditions in which there was enough information, we conclude that high stress levels, whether intrinsic (triggered by the cognitive challenge) or extrinsic (induced by conditions completely unrelated to the cognitive task), tend to facilitate Pavlovian conditioning (in a linear-asymptotic manner), while being deleterious for spatial/explicit information processing (which with regard to intrinsic stress levels follows an inverted U-shape effect). Moreover, after reviewing the literature, we conclude that all selected factors are essential to develop an integrative model that defines the outcome of stress effects in memory processes. In parallel, we provide a brief review of the main neurobiological mechanisms proposed to account for the different effects of stress in memory function. Glucocorticoids were found as a common mediating mechanism for both the facilitating and impairing actions of stress in different memory processes and phases. Among the brain regions implicated, the hippocampus, amygdala, and prefrontal cortex were highlighted as critical for the mediation of stress effects.

## 1. INTRODUCTION

Nowadays, there is great consensus in the literature that stress is a potent modulator of cognitive function in general, and more precisely, of learning and memory processes McEwen and Sapolsky [1]; de Kloet et al. [2]; Lupien and Lepage [3]; Sandi [4, 5]; Diamond et al. [6]; Fuchs et al. [7]; Joëls et al. [8]; Shors [9]. Although stress effects are frequently regarded as deleterious to cognitive function, very intensive work during the past decade is delineating a great complexity, both in the nature of interactions between stress and memory functions and in their outcome. In addition to the overemphasized negative side of stress on brain and behavior, there are many instances in which neural function and cognition are either facilitated by stress (de Kloet et al. [2]; Joëls et al. [8]), or even not affected (Warren et al. [10]; Beylin and Shors [11]).

There have been several successful attempts to make sense of the confusion in the literature. By focusing on specific explanatory factors, different authors have successfully provided integrative and clarifying views of the impact of stress on memory function. For example, a great deal of the variability can be explained by the “intensity” of the stressor, either if the dosage reflects its physical characteristics (Cordero et al. [12]) or internal hormonal reactions (Baldi and Bucherelli [13]; Conrad [14]; Joëls [15]). The most general view is that stress—or stress hormones—levels induce inverted U-shaped dose effects in learning, memory, and plasticity (Baldi and Bucherelli [13]; Conrad [14]; Joëls [15]), although linear effects have also been proposed (Diamond [16]). A second important factor that has been emphasized is stress “duration,” with distinct effects frequently induced by *single* versus *repetitive*—or chronic- stress—or stress hormones activation-, and not only at the cognitive level, but also when evaluating brain structure and function (Sandi and Loscertales [17]; Pinnock and Herbert [18]; Pecoraro et al. [19]; Joëls et al. [8]). A third important factor that has been particularly highlighted by Roozendaal [20, 21] as relevant in this context is the memory phase at which stress acts. After reviewing the literature, Roozendaal [20, 21] has proposed opposing effects for stress—and stress hormones activation—during the phases of *consolidation* (generally facilitating) and *retrieval* (generally impairing) of information. A fourth factor that should be mentioned is psychological factors, notably stressor *controllability* and *predictability* that are well known to be key mediators of the psychophysiological impact of stress (Mineka and Hendersen [22]; Das et al. [23]). Convergent evidence indicates that experiencing uncontrollable—as opposed to controllable—stress has deleterious effects on further information processing (Maier and Watkins [24]). A fifth factor that seems to count for the outcome of stress in memory function is the importance of taking into account the existence of individual differences when trying to make sense of the literature on stress and memory, with *gender* appearing as a very highly important modulator of such interactions (Luine [25]; Bowman et al. [26]; Shors [27]). Finally, a sixth factor that has been identified as certainly relevant to understand how stress affects cognition is the relevance of the context in which stress—or stress hormones activation—is experienced, that is, whether stress is, or is not, *contingent* to the particular information processing under study (Sandi [28]; de Kloet et al. [2]; Joëls et al. [8]).

Despite the usefulness of the above-mentioned factors, a systematic view that integrates all the complexity (or at least much of it) of the apparently discrepant actions of stress in cognition is still lacking. Although not so ambitious as to try to develop a comprehensive model including all the factors highlighted above, our goal here is to come up with an integrative model that incorporates several of them along with new proposed factors. More specifically, our goal is to organize the literature among those selected factors to eventually provide integrative answers to the question: “what does it count for the outcome of stress interaction with memory function”? Finally, we will evaluate whether such integrative effort helps understanding better stress effects on memory function than other more reductionistic approaches already available in the literature. We should also state that the goal of this review is to discuss studies from the literature that help illustrating the mediating influence of the selected factors (see above) to understand the nature of stress actions on memory function. By no means, we attempt to include here an exhaustive account of a large number of studies that have proliferated in recent years. In addition, each subsection includes a brief account of the main neurobiological mechanisms proposed to account for the different effects of stress in memory function.

## 2. FACTORS SELECTED TO ANALYZE STRESS AND MEMORY INTERACTIONS

We should emphasize that the revision and potential final model will account for the impact of stress in adult male rodents according to the following factors.

**Figure 1:**
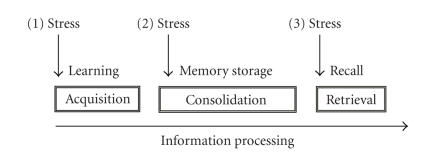
Diagram depicting the relevance of specifying timing of stress with regards to different memory phases. If stress (1) is given before learning (acquisition of information), it can potentially affect all cognitive phases involved in memory function; that is, acquisition, consolidation, and/or retrieval. However, if acquisition is already affected, that would be the main conclusion to extract from the particular experiment. If stress (2) is experienced after learning, any effect observed in retention could now be due to an impact of stress on either consolidation or retrieval, but any effects on acquisition can be discarded. However, effective treatments given at this time point normally disrupt the process of memory storage, instead of retrieval, which can be further tested by given the treatment at later time points (at a different—or outside the—consolidation phase) and assess whether recall is then also affected. If stress (3) is delivered before the recall test, it should just normally affect the retrieval processes. However, a note of caution should be mentioned depending on how close the retention test is applied with regards to training, since consolidation mechanisms are increasingly recognized to last longer than previously hypothesized and, therefore, this type of manipulation could influence both consolidation and retrieval processes. Research on this field should take into account this complexity and apply the necessary controls to ascertain which phase and mechanisms of the information processing is affected by the stress procedure under study.

(1) Source of stress: we will introduce a new factor, the source of stress, and emphasize its utility to understand the diversity of stress and memory interactions. It makes reference to the origin of stress with regard to the cognitive task. In a way, it is related to the above-mentioned factor *contingency to the contex* (de Kloet et al. [2]; Joëls et al. [8]), but it includes a more explicit nomenclature that hopefully will help clarifying the concept. More precisely, this factor classifies stress as either *intrinsic* (if stress is originated by elements related to the cognitive task) or *extrinsic* (if stress is originated by conditions completely unrelated to the cognitive task, i.e., in the outside world, and ideally occurring temporally dissociated from such task, i.e., either before or afterwards).

(2) Stressor duration: this factor makes reference to the length of stress. The differential effects of *acute* versus *chronic* (with some *subchronic* versions) stress have concentrated great interest in the field. In addition to the relevance to cognitive function, this factor is essential when evaluating the neural mechanisms whereby stress affects cognition.

(3) Stressor intensity: stressors can vary throughout a very wide range of intensities. Even though oversimplifications can have the drawback of being too superficial, for the sake of clarity, we will just use the categories of *low*, *medium*, *high* (and occasionally *very high*) intensities. Not surprisingly, very high (e.g., a clear life threat, such as a being in a combat) and mild (e.g., novelty exposure) stressors seem to have distinct effects on cognitive function (Cordero et al. [12]; Joëls et al. [8]). Importantly, since conspecifics frequently show marked individual differences in stress reactivity (Márquez et al. [29]), measuring individual behavioral and physiological responses to a particular stressor would be the ideal approach when trying to determine the actual stress magnitude experienced by each experimental subject. When such approach is not possible, it is important to be systematic in the gradation of the amount of stressor applied to the different animals, ideally including at least three different intensities.

(4) Stressor timing with regard to memory phase: this factor makes reference to the time when stress is experienced with regard to a particular memory phase. Memory phase stands for the type of the information process that is linked to stress. Generally, three phases are distinguished: acquisition (the learning process), consolidation (memory storage), and retrieval (access to stored information) of information (see Figure 1). As noted above, stress and stress mediators appear to exert opposing effects in consolidation and retrieval (Roozendaal [20, 21]; but see de Kloet et al. [2]; Joëls et al. [8]).

(5) Learning type: an additional key factor is the type of the learning process that is evaluated (i.e., implicit/nondeclarative learning, explicit/declarative learning, nonassociative learning, etc.). Although there are different typologies of memory involving a variety of subtypes (Nelson et al. [30]; Squire and Zola [31]; Verfaellie and Keane [32]; Eichenbaum [33]; Moscovitch et al. [34]), this review will focus on a main dichotomy between a type of *implicit* memory processes, Pavlovian conditioning, and spatial types of learning (when reviewing the animal literature) as models for *explicit* memory processes.

Even though we will occasionally mention relevant studies in other species (notably, in humans), this is a review about the rodent literature. Importantly, we will not include as analytic factors two of the probably most important ones among the large list proposed above: (i) psychological factors, like controllability and predictability; (ii) individual differences in the vulnerability and response to stress. Whenever the effect of stress “from outside the context” is applied, we review studies that applied “uncontrollable” stressors and deliberately excluded the few studies that examined the role of “controllable” ones. Concerning the issue of individual differences, we concentrate on the studies performed in adult (but not old) male rodents. We have decided not to tackle here the role of gender, since there are still not enough studies performed in female rodents for each of the factor conditions included in the study. Moreover, we should clarify that we will not deal here with studies in which the impact of stress was evaluated from a developmental point of view, such as for example how pre- or postnatal stress affects cognition in adulthood. Typically, the type of stress whose effects we will examine is stress closely associated with the cognitive challenge under study/discussion, and therefore normally experienced from a few minutes to normally 1-2 days either before or after a particular memory phase.

We have selected the factor “source of stress” as the guiding line to structure this review. We hypothesize that intrinsic stress facilitates learning and memory processes, whereas “extrinsic” stress will normally have the opposite impairing effects. Although differing in some ways, this hypothesis shares some commonalities with the proposal formulated by Joëls et al. [8] stating (page 154):

“…*that stress will only facilitate learning and memory processes: (i) when stress is experienced in the context and around the time of the event that needs to be remembered, and (ii) when the hormone and transmitters released in response to stress exert their actions on the same circuits as those activated by the situation, that is, when convergence in time and space takes place*…”

In the following pages, relevant studies from the literature will be first classified depending on whether the source of stress is intrinsic or extrinsic to the memory task, and then will be analyzed according to each of the other four factors selected for the analysis (stressor duration, stressor intensity, timing with regard to memory phase, and learning type).

## 3. THE IMPACT OF ACUTE INTRINSIC STRESS ON MEMORY FUNCTION

As stated above, intrinsic stress makes reference to those situations in which stress is either elicited by, or directly associated with, the cognitive experience. Let us first consider how the factors highlighted above account for intrinsic stress conditions in order to define the whole extent of settings that will be discussed here.

- Stressor duration: although intrinsic stress (or stress linked to a cognitive experience) can be experienced both acutely and chronically, to our knowledge, no study to date has systematically studied how chronic activation of stress systems during learning experiences contributes to the different phases involved in memory processes (from learning acquisition to memory consolidation, relearning, reconsolidation, retrieval of information, etc.). Therefore, the evaluation resulting from this review for intrinsic stress will only account for *acute* (not chronic) situations in which a memory is formed from a stressful learning experience.
- Stressor intensity: whenever possible, we will consider the whole range of stress intensities: *low*, *medium*, *high*, and occasionally *very high*.
- Stressor timing with regard to memory phase: as noted above, to be considered within the category of intrinsic stress, stress should be linked to a particular cognitive challenge. This could be either a learning challenge or a retrieval challenge. Although several studies have focused on the role of intrinsic stress linked to the learning phase, to our knowledge, no study has systematically studied how stress elicited by the retrieval experience accounts for the effectiveness of the retrieval process. Therefore, the evaluation resulting from this review for intrinsic stress will only account for *learning* (not retrieval) processes. Importantly, stressful learning experiences might affect potentially the *acquisition* and/or *consolidation* of information. We will examine separately both memory phases.
- Learning type: as mentioned above, this review focuses in *Pavlovian conditioning* (as representative of implicit learning) and *spatial learning* (as representative of explicit learning). Since there are examples in the literature for both learning types, the discussion here will include and compare the impact of intrinsic stress upon both learning types.

Summarizing, in this subsection, we will evaluate how stress (in a dose-response fashion) triggered by a learning challenge (therefore, an acute condition) affects memory (both implicit and explicit types of memory) function.

Emotionally arousing experiences are better remembered than more neutral ones (Cahill and McGaugh [35]; Sandi [28]; McGaugh [36]). The emotional reaction can range from a mild activation to a strong stress response, and therefore, stress can be regarded as a critical component within the framework of the emotional modulation of memory. The evolutionary advantage of ensuring the future recalling of specific aversive stimuli and/or the successful strategies developed once by the individual to cope with such aversive stimuli is clear. The rapid identification of already experienced dangers, as well as the ability to enhance the speed and accuracy of behavioral reactions to threats, provides the individual with better survival possibilities if faced with similar dangerous circumstances in the future. Predictably, this will, in turn, revert on enhanced reproductive success.

Classically, research attempts addressed to characterize the facilitating effects of stressful learning on memory function have emphasized the role of stress-induced mechanisms on the *consolidation* of the information acquired during such stressful event (Roozendaal [20, 21]). However, enhanced memories resulting from stressful learning situations can also be due, on a first instance, to an effect of stress on the *acquisition* of information. This can be achieved by altering a variety of psychobiological functions (such as attention, motivation, sensory processing and integration, and motor function) that are known to be both sensitive to stress and able to modulate learning processes. Although these latter processes have been less explored in research programs, we will review here the contribution of stress to the spectrum of information encoding including both the storage—consolidation—and acquisition of information.

### 3.1. Effects of intrinsic stress on the consolidation of information

The effects of arousing or stressful experiences on memory consolidation—as well as the potential mediating mechanisms—have received much attention over the past decades (Sandi [28], Roozendaal [20, 21]; Conrad [14]; McGaugh and Roozendaal [37]; Richter-Levin and Akirav [38]; McGaugh [36]; de Kloet et al. [2]; Joëls et al. [8]).

Different approaches have been successfully undertaken to assess whether the degree of stress experienced during learning might be related to the strength of the memory that is formed. One of those approaches (reviewed below) is based on the manipulation of the intensity of the stressor used as the unconditioned stimulus (US) in a particular task, to subsequently evaluate whether any correlation can be observed between posttraining levels of stress hormones and the degree of memory displayed by the animals.

#### 3.1.1. Pavlovian conditioning

**Figure 2:**
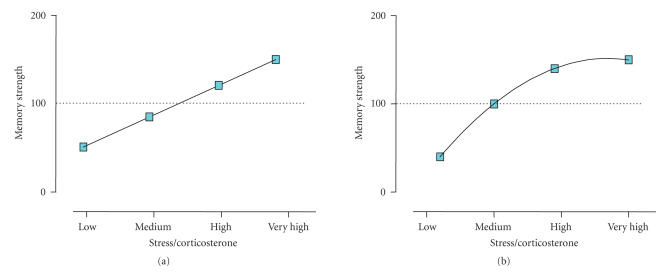
Impact of “intrinsic” stress on memory consolidation. Figures representing the linear (a) and linear-asymptotic (b) relationship between stress intensity (either defined by the stressor or by the physiological response indexed by the glucocorticoid corticosterone) experienced during the consolidation period (provided learning has taken place during the previous learning phase) and the strength of the memory formed.

Typical examples of this type of studies are those involving different shock intensities in fear conditioning tasks. Experiments performed in rats with the contextual fear conditioning task, involving groups that received different shock intensities (0.2, 0.4, and 1 mA), observed a direct relationship between the stressor intensity experienced at training and the level of freezing displayed by animals at the testing session (Cordero et al. [12, 39]; Merino et al. [40]). Similar shock-dependent effects on auditory fear conditioning have also been described for mice (Laxmi et al. [41]; Anagnostaras et al. [42]). Therefore, these data support the existence of a linear relationship between stressor intensity and the strength of fear conditioning memory formed (see Figure 2(a)). Although difficult to study for obvious ethical reasons restricting the magnitude of stress that can be delivered to animals, one would expect that the dose-dependent linear relationship would achieve an asymptotic, or ceiling effect, after certain stressor intensity is achieved (see Figure 2(a)). To our knowledge, no study has found evidence for impaired memory consolidation for fear conditioning at very high stress conditions. If we consider the normal range of experiences to which experimental animals are submitted in the laboratories worldwide, a stressor intensity-dependent linear relationship seems to account for the effects of stress in the formation of fear memories (Rau et al. [43]).

*Conclusion*

A linear relationship is proposed for the impact of different stress intensities on the consolidation of fear conditioning, with an asymptotic wave form for high-to-very-high stress intensities (Figure 2).

*Neurobiological mechanisms*

Interestingly, posttraining corticosterone levels showed a positive correlation with the strength at which fear conditioning is established into a long-term memory (Cordero et al. [12, 39]; Merino et al. [40]) (see Figure 2(a)). A causal role for a central action of corticosterone through glucocorticoid receptors has been supported by two complementary types of studies. First, posttraining administration of corticosterone (either peripherally or centrally) facilitates memory consolidation for both contextual (Pugh et al. [44]; Cordero and Sandi [45]; Revest et al. [46]) and auditory fear conditioning—an effect that was dose-dependent and specific for the conditioned tone (Hui et al. [47]). Second, inhibition of either training-induced corticosterone release (Cordero et al. [39]; Fleshner et al. [48]) or central antagonism of the glucocorticoid, but not mineralocorticoid, receptors (Cordero and Sandi [45]) inhibited the strength of the fear memory formed. Microinfusion of a glucocorticoid receptor antagonist in the basolateral nucleus of the amygdala (BLA) and ventral hippocampus was also found to interfere with long-term memory of contextual fear (Donley et al. [49]).

Recent evidence (Revest et al. [46]) has implicated the MAPK pathway within the hippocampus in the increase in contextual fear conditioning induced by glucocorticoids. Another research line has implicated the neural cell adhesion molecule (NCAM) in the stressor intensity-dependent effects on fear memory formation (Merino et al. [40]). Moreover, the enhancing effect of corticosterone on memory consolidation of auditory-cue fear conditioning requires posttraining noradrenergic activity within the BLA (Roozendaal et al. [50]) and is associated with increased expression of CRH mRNA in the amygdala (Thompson et al. [51]).

#### 3.1.2. Spatial learning

In the spatial learning water-maze task, a similar dose-dependent phenomenon for stress regulation of memory consolidation has been described. In this case, stress intensity was varied by manipulating the temperature of the pool water during the acquisition phase (Sandi et al. [52]). Rats learning the task at a water temperature of 19°C showed a greater retention of the platform location on the second day of training than rats trained at 25°C. Again, a relationship was found between the strength of memory and corticosterone levels displayed by rats after the first training session, with rats trained on the experimental conditions that led to a stronger and longer-lasting memory (i.e., at 19°C) showing the highest circulating hormone levels. These hormonal data indicated that training at 19°C is more stressful than training at 25°C. Moreover, performance of rats trained at 25°C, but not at 19°C, was improved by peripheral injections of corticosterone given immediately after each training session. Therefore, these results further support the existence of a linear facilitating effect of stress on memory consolidation, with increasing glucocorticoid levels during the posttraining period reinforcing the strength of memory up to an asymptotic or ceiling effect (Figure 2(b)).

*Conclusion*

A linear asymptotic relationship is also proposed for the impact of different stress intensities on the consolidation of spatial learning, with ceiling performance already achieved for high stressor intensities (Figure 2).

*Neurobiological mechanisms*

Several examples in the literature support a wider range for the dose-response relationship between glucocorticoid levels and consolidation of spatial learning. Detrimental effects of low glucocorticoid levels in learning and plasticity processes have been largely documented in different tasks. For example, either metyrapone (an inhibitor of glucocorticoid synthesis and release) administration or adrenalectomy-inhibited special memory in a variety of mazes, including the water maze (Oitzl and de Kloet [53]; Roozendaal et al. [54]), radial arm maze (Vaher et al. [55]), and Y-maze (Conrad et al. [56]). In addition, blocking the activation of glucocorticoid receptors with the GR antagonist RU-38486 impaired spatial memory in the water maze (Oitzl and de Kloet [53]; Roozendaal and McGaugh [57]). Interestingly, similar results have also been obtained in humans; with metyrapone administration enhancing the rate of forgetting on a declarative memory task (Lupien et al. [58]). Glucocorticoid receptors can affect transcription both through DNA binding-dependent and independent mechanisms. Using male mutant mice in which homodimerization and DNA binding of the glucocorticoid receptor is largely prevented (GR(dim/dim)) while protein-protein interactions still can take place (Oitzl et al. [59]), the facilitating effects of corticosterone on spatial memory were shown to depend on DNA binding of the glucocorticoid receptor.

Interestingly, the activation of ERK2 in the hippocampus and the amygdala differs in animals trained at 19°C and 25°C. In the dorsal CA1, training induced an increased phosphorylation of ERK2 only in animals that had learned the task (irrespective of the level of stress). In contrast, in the amygdala, activation of ERK2 was found only in animals that learned the task well under high levels of stress (19°C) (Akirav et al. [60]).

Adrenergic mechanisms have also been implicated in the consolidation of spatial memories. Water-maze learning also triggers the release of adrenergic (adrenaline and noradrenaline) hormones. Mabry et al. [61] showed that plasma adrenaline and noradrenaline levels in young adult rats submitted to water swimming are correlated with water temperature, with 20°C inducing higher glucocorticoid hormonal levels than 25°C. Interestingly, good and bad learners in the water maze at 25°C have been suggested to differ in their task-induced endogenous activation of adrenergic hormone release (Cahill et al. [62]), since posttraining administration of the beta-adrenergic antagonist propranolol specifically impaired the good retention levels showed 24 hours after training by “good learners,” without affecting performance in “poor learners.” These findings were interpreted as the possible involvement of posttraining adrenergic activation in modulating memory consolidation processes after emotionally stressful events. Interestingly, direct injections of propranolol into the BLA cause retrograde amnesia in the same water-maze task (Hatfield and McGaugh [63]). Several findings in humans have provided support for the hypothesis that enhanced memory for emotionally arousing events depends critically on posttraining adrenergic modulation (Cahill et al. [64]; Southwick et al. [65]). The fact that the degree of activation of the noradrenergic system following training predicts retention performance supports the view that the noradrenergic system within the amygdala plays a central role in memory consolidation. In fact, this phenomenon is circumscribed within more general evidence that the modulation of long-term storage of an emotionally arousing event involves an important activation of the noradrenergic system within the amygdala (McGaugh [36]). Moreover, the dopaminergic system in the BLA has been suggested to be critically involved in memory modulation induced by the noradrenergic system (Lalumiere and McGaugh [66]).

### 3.2. Effects of intrinsic stress on the acquisition of information

Although the facilitating role of stress on consolidation has been emphasized for many years, less attention has been paid to the effects of intrinsic stress on acquisition of information. One of the main reasons for this reduced attention is the variability in the length and characteristics of learning protocols, some including one-trial training procedures and others involving multiple learning trials and even sessions. Such diversity makes it difficult to reach conclusions as to whether it is the acquisition of information that is affected by prior stress, working memory processes, or other types of mechanisms. Anyhow, more recent work raises the possibility that stress effects on acquisition might also underlie the potentation of long-term memory observed when learning under stress.

#### 3.2.1. Pavlovian conditioning

**Figure 3:**
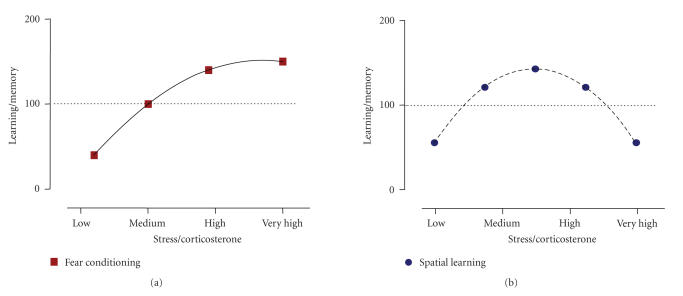
Impact of “intrinsic” stress on learning acquisition. Figures representing the linear-asymptotic—typical for fear conditioning—(a) and inverted U-shape—typical for spatial learning—(b) relationships between stress intensity (either defined by the stressor or by the physiological response indexed by the glucocorticoid corticosterone) experienced during the learning period and the degree of learning and memory acquired.

Such possibility is quite clear for fear conditioning. When we talk of a linear relationship between shock intensity and long-term memory, we cannot neglect the fact that such linear relationship already exists during the conditioning phase between shock intensity and behavioral reactivity (Figure 3(a)). High shock intensities are typically followed by higher freezing responses than those displayed to lower shock intensities (Cordero et al. [12]; Merino et al. [40]; Laxmi et al. [41]).

However, and although in many occasions mechanisms operating during acquisition will already be key for the strength of the long-term memory formed, we cannot disregard the existence of an acquisition-independent dose-dependent effect for stress and consolidation. The fact that some of the treatments addressed to interfere with the cognitive actions of stress systems (such as, e.g., glucocorticoid administration, or interference experiments based on either corticosterone synthesis inhibition (Cordero et al. [39]) or antagonism of glucocorticoid receptors (Cordero and Sandi [45])) did not affect with the after-shock freezing response but did impair long-term memory reinforces the view that those physiological stress systems show a dose-dependent effect on memory consolidation. The possibility that initial encoding is also affected for such treatments should be more systematically addressed, and would require, for example, fine behavioral analyses during the conditioning processes as well as testing animals in the task at very short time intervals after conditioning.

*Conclusion*

A linear asymptotic relationship is observed for the impact of different stressor intensities in performance during the acquisition of fear conditioning (Figure 3(a)).

#### 3.2.2. Spatial learning

The example given above for water-maze training at different water temperatures (Sandi et al. [52]) was a spaced learning protocol extended over a few consecutive days. It presented the advantage that by just giving a few training trials per day, groups of animals trained at either 19°C or 25°C water temperature did not differ in their performance on the first training session. However, clear differences were observed in their retention levels from the second training day on, with rats trained at 19°C showing better performance than animals that had been trained at 25°C. This effect was already on the first trial of the second training day; indicative of differences in the strength of memory raised during the consolidation period. The same effect was observed in animals that had been trained at 25°C followed by an injection of corticosterone. Altogether, those results reinforced the view of a facilitating action of stress and glucocorticoids (and note also that evidence is discussed above for adrenergic mechanisms) on consolidation mechanisms.

However, in spatial learning tasks, there are a few documented cases in which learning under different stress levels can have an immediate impact on the rate of learning. By using a modified version of the Morris water maze task that consists in a massed training protocol (1 hour of training in 1 day) that generates long-term spatial learning, Akirav et al. [60] showed that rats trained at 19°C and 25°C already differ in their acquisition rate during the training session. Rats trained at 19°C displayed shorter latencies to find the hidden platform than rats trained at 25°C. Interestingly, animals trained at 25°C could be split into two groups, one that performed as well as the 19°C trained animals and another that performed poorly (i.e., showed longer latency to reach the hidden platform in the water maze), with differences in performance at 25°C apparently being related to the anxiety trait of animals (Herrero et al. [67]).

Interestingly, Akirav et al. [60] also reported that differences in animals' learning curves correlated with corticosterone levels, with higher hormone levels observed in rats trained at 19°C. In a subsequent study, Akirav et al. [68] explored the role of glucocorticoids on learning and memory processes in the same training paradigm. Rats injected with the corticosterone synthesis inhibitor metyrapone (50 or 75 mg/kg, but not 25 mg/kg) showed an impaired learning rate at 19°C, as well as impaired spatial memory. Conversely, rats injected with corticosterone (10 mg/kg, but not 25 mg/kg) at 25°C showed both a better learning rate and better subsequent retention. Therefore, these data also strongly implicate corticosterone in the level of acquisition of spatial learning. They also indicate that there is a ceiling effect for the facilitating actions of corticosterone during acquisition of spatial information, since the dose of 10 mg/kg facilitated learning, whereas the higher dose of 25 mg/kg did not. This finding should be considered cautiously, since the dose of 25 mg/kg might, in fact, induce more pharmacological than physiological levels of the steroid, but it could also suggest the existence of biphasic effects of stress and glucocorticoids in learning acquisition. However, we should also note that rats trained at 25°C that showed a poor performance showed significantly enlarged corticosterone responses (Akirav et al. [60]). These results, together with the higher corticosterone levels displayed by poor performers trained 19°C (see above), further suggest the existence of an inverted U-shaped relationship between corticosterone levels and performance at training (Figure 3(b)).

Such possibility (the existence of an inverted U-shape between stress levels and learning acquisition for spatial tasks) is reinforced by a previous study (Selden et al. [69]) that showed impaired spatial learning in animals trained at 12°C, a highly stressful condition for the animals. Such impairment was prevented by noradrenaline depletion in the dorsal noradrenergic bundle (ceruleocortical pathway), which only affected performance under such stressful condition, but not in animals trained at a higher temperature (26°C).

*Conclusions*

The reviewed data on spatial learning supports the view that the effectiveness of acquisition throughout a continuum of stress and/or corticosterone levels generally follows an inverted U-shaped function; the lower performance associated with very low and very high levels, and the optimal performance with intermediate stress levels (see Figure 3(b)).

*Neurobiological mechanisms*

How could stress systems activated by the training experience affect the learning rate? Whereas an immediate effect of noradrenergic systems in acquisition and performance can be explained by their well-known actions in modulating attention (Selden et al. [69]), explaining online actions of glucocorticoids might not be so straightforward. Typically, glucocorticoid actions were believed to be genomic, with activated corticosteroid receptors being able to modulate the transcription of a large number of genes (Beato and Sanchez-Pacheco [70]; Datson et al. [71]). Such effects are of slow appearance, and therefore cannot explain the described differences in performance throughout the massed spatial training protocol due to different stress conditions (water temperatures). However, increasing evidence supports the existence of rapid effects of glucocorticoid through nongenomic mechanism (Sandi et al. [72, 73]; Karst et al. [74]; for reviews see Makara and Haller [75]; Dallman [76]; Tasker et al. [77]). Glucocorticoids could rapidly modulate cognition through their ability to rapidly enhance extracellular glutamate levels, as shown in the hippocampus and prefrontal cortex, both during stress (Lowy et al. [78]; Moghaddam et al. [79]) and following a peripheral injection of corticosterone (Venero and Borrell [80]). In connection with these fast actions of corticosterone on glutamate release, Karst et al. [74] have recently reported that stress levels of corticosterone, by interacting with the mineralocorticoid receptor (MR), can rapidly enhance the frequency of miniature excitatory postsynaptic potentials in hippocampal CA1 pyramidal neurons and to reduced paired-pulse facilitation. Given that the MRs have been traditionally regarded as the mediators of tonic actions of glucocorticoids, it is important to mention recent evidence suggesting that MR protein expression in the brain can be rapidly regulated by changes in corticosteroid levels (Kalman and Spencer [81]). In addition, some of the rapid glucocorticoid actions can also be mediated through interactions of glucocorticoid metabolites on the gamma-aminobutyric acid (GABA) system (Strömberg et al. [82]).

In addition, the intriguing possibility that glucocorticoids could also rapidly affect the density and morphology of dendritic spines in CA1 pyramidal neurons within 1 hour has been recently put forward (Komatsuzaki et al. [83]). Dendritic spines are essential for information processing, and therefore for memory formation. Because the presence of the protein synthesis inhibitor cycloheximide did not block the effect of the synthetic glucocorticoid dexamethasone, the authors suggest that such rapid morphological changes are probably nongenomic. Moreover, this study presented evidence for the localization of the classical GR in synaptosomal fractions enriched in postsynaptic membranes, suggesting a possible action site of dexamethasone at spines. However, these findings were obtained in hippocampal slices, and therefore the validity for the in vivo situation still remains to be established.

## 4. THE IMPACT OF ACUTE EXTRINSIC STRESS ON MEMORY FUNCTION

We will deal here with those situations in which stress experienced by the individual is not related to the cognitive task, but is elicited by other circumstances happening either before or after the mnemonic experience (i.e., stress comes from “the outside world”). This condition, that we term extrinsic stress, resembles the concept of “out-of-the-learning context” proposed by other authors (de Kloet et al. [2]; Joëls et al. [8]). At difference to intrinsic stress for which there were not studies exploring the contribution of chronic conditions, there are many examples in the literature devoted to explore the effects of extrinsic stress, both for acute and chronic conditions. Therefore, we will deal with these two very different phenomena in separate subsections, starting here with those referring to acute extrinsic stress. As we did for intrinsic stress, we will first consider which of the factors selected for the current analysis (see above) account for acute extrinsic stress conditions.

- Stressor duration: as noted above, both *acute* and chronic situations are well documented in the literature. In this subsection, we deal with *acute* stress.
- Stressor intensity: although, hypothetically, the impact of a range of stressor intensities on cognitive performance could be studied, most reports that investigated extrinsic stress conditions generally just apply a single stressor intensity. Whenever possible, we will grade the stressor intensities delivered by the studies according to the same range as above: *low*, *medium*, *high*, and *very high*.
- Stressor timing with regard to memory phase: extrinsic stress can be delivered either before (acquisition) or after (consolidation) *learning*, or before *retrieval*. For Pavlovian conditioning, there are examples in the literature related to acquisition and consolidation, whereas for spatial learning the available examples are related to acquisition and retrieval. We will review below each of these memory phases separately, as appropriate.
- Learning type: we will deal with examples for both *Pavlovian conditioning* and *spatial learning*.

Summarizing, in this subsection, we will evaluate how acute stress (at different intensities) experienced outside the learning challenge affects memory (both implicit and explicit types of memory) function.

### 4.1. Effects of acute extrinsic stress on the acquisition of information

#### 4.1.1. Pavlovian conditioning

There are many examples in the literature in which prior exposure to acute stress affects subsequent learning in Pavlovian conditioning tasks. The topic has been addressed recently in several reviews (Shors [9, 27]).

Shors and collaborators have extensively illustrated that stress experienced before training consistently facilitates eyeblink conditioning in male rats of different strains (Shors et al. [84]; Servatius and Shors [85]; Shors and Servatius [86]; Wood and Shors [87]; Beylin and Shors [11]; Shors [88]). Interestingly, stressors of medium intensity displayed no effect on conditioning, with high-to-very-high stressful conditions, (typically a restraint-tailshock procedure, unpredictable and uncontrollable, adapted from the “learned helplessness” paradigm) being required to potentiate this learning process (Shors and Servatius [86]; Beylin and Shors [11]). The enhancement of learning by prior acute high stress was observed during classical eyeblink conditioning of both hippocampal-dependent and independent learning tasks. It could be triggered within minutes of the stressful event and lasted for days.

Acquisition of fear conditioning has also been shown to be highly susceptible to modulation by prior stress exposure. Prior shock exposure has been shown to greatly enhance subsequent contextual fear conditioning in a different context (Fanselow and Bolles [89]; Fanselow et al. [90]). Likewise, previous exposure to an acute restraint session increased contextual fear conditioning (Cordero et al. [91]; Rodriguez Manzanares et al. [92]). Moreover, using the BALBc strain of mice, Radulovic et al. [93] showed that restraint stress, in addition to its facilitating effects in contextual conditioning, it also enhances auditory-cued fear conditioning processes.

*Conclusions*

**Figure 4:**
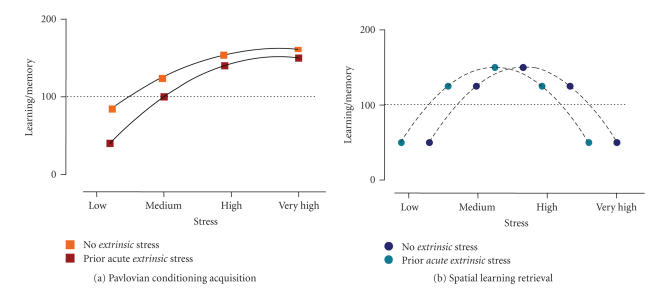
Impact of “acute extrinsic” stress on memory function. Figures representing how extrinsic stress can affect the linear-asymptotic (a) and inverted U-shape (b) relationships depending on the intrinsic stress of each of the learning tasks. Note that, according to the available knowledge in the literature, this model accounts for the “acquisition” of Pavlovian conditioning (a) and for the “retrieval” of spatial information (b). In both conditions, extrinsic stress is proposed to displace to the left the relationship between stressor-related relationship and performance (however, this displacement in the case of the inverted U-shape in (b) has only been described for the right part of the curve).

Therefore, high extrinsic stress facilitates Pavlovian fear conditioning. Although a systematic study should be performed, we propose that extrinsic stress shifts the dose-dependent impact of the unconditioned stimulus to the left (see Figure 4(a)).

*Neurobiological mechanisms*

The enhancement of both types of Pavlovian learning discussed here, eyeblink conditioning (Beylin and Shors [94]) and fear conditioning (Cordero et al. [91]), involves glucocorticoids. In the eyeblink conditioning task, endogenous glucocorticoids were shown to be necessary and sufficient for transiently facilitating acquisition of new associative memories, and necessary but insufficient for persistently increasing their acquisition after exposure to acute stress (Beylin and Shors [94]). In the contextual fear conditioning task, animals that had been previously submitted to a single restraint session showed increased corticosterone levels following training, which suggested that increased glucocorticoid release at training might be implicated in the mechanisms mediating the memory facilitating effects induced by prior stress experiences (Cordero et al. [91]).

Anxiety mechanisms have also been related to the enhancing effects of prior stress in Pavlovian conditioning. Recent evidence provided by Bangasser et al. [95] implicated the bed nucleus of the stria terminalis (BNST) in the facilitating effects induced by stress in eyeblink conditioning. Interestingly, in humans, high degrees of trait or state anxiety have also been linked with increases in eyeblink conditioning (reviewed by Shors [9]). In the restrain stress-induced facilitation of fear conditioning, changes in GABAergic mechanisms in the amygdala have been implicated, that is, stress was shown to induce an attenuation of inhibitory GABAergic control in the BLA, leading to neuronal hyperexcitability and increased plasticity (Rodriguez Manzanares et al. [92]).

#### 4.1.2. Spatial learning

The same acute stress procedure that was repeatedly shown by Shors et al. (see above) to facilitate eyeblink conditioning was found not to have any effect in performance during learning in the Morris water maze (Warren et al. [10]; Healy and Drugan [96]; Kim et al. [97]) (but note that in one of these studies, animals were subsequently impaired in their retention levels for the platform location (Kim et al. [97])). Similarly, exposure to cat stress before training did not affect the rate of acquisition of platform location in a radial arm water maze (Diamond et al. [98]) (but note again that this pretraining stress resulted in impaired spatial memory when tested 24 hours later). Furthermore, this lack of effect does not seem to be restrictive to stressful water maze tasks. By using a nonspatial object-recognition memory task and the same inescapable restraint and tail-shock stress procedure as mentioned above, similar results have been reported by Baker and Kim [99]. Rats stressed before being exposed to the task showed normal memory when tested 5 minutes after first exposure to objects, but were impaired when tested 3 hours afterwards. Control rats display a preference for a novel object (over a familiar one) when they are tested at different time delays (5 minutes and 3 hours). As opposed to these unstressed controls, at the 3-hour posttraining test, stressed animals spent comparable time exploring novel and familiar objects.

However, we should mention that work in mice has pointed out the importance of individual differences in the impact of acute extrinsic stress on spatial learning. Francis et al. [100] evaluated the effect of daily exposure to uncontrollable footshocks before spatial orientation. They found that such treatment did not affect the acquisition or performance of this response in three strains (DBA/2J, C57BL/6J, BALB/cByJ), but provoked a modest disruption of reversal performance in DBA/2J mice and markedly impaired reversal performance in BALB/cByJ mice. The authors emphasized the importance of individual differences in the susceptibility to stress and speculated that uncontrollable stress would not disturb response-outcome associations, but may induce a perseverative response style. Therefore, a potential effect of stress in reversal learning cannot be neglected.

*Conclusion*

Learning new spatial associations (i.e., when an individual is confronted for the first time to find a reward in a particular spatial setting) is a process highly resistant to the effect of prior stress (even when involving high to very high stress conditions). However, the more flexible process of reversal learning (i.e., when there is a change in the location of a reward in a particular spatial setting, from a former place to a new one, and the individual is then confronted to reverse the strategy) to find a reward seems to be more vulnerable to disruption by prior stress.

### 4.2. Effects of acute extrinsic stress on the consolidation of information

#### 4.2.1. Pavlovian conditioning

There are only a few examples in the literature focusing on the impact of posttraining acute stress on consolidation of Pavlovian conditioning, and the results are less homogeneous than for acquisition.

Using the eyeblink conditioning paradigm in rats, Beylin and Shors [11] showed that the same high intensity stressor that facilitates conditioning when applied before training does not influence further retention levels when it is delivered after animals have been conditioned.

Social isolation stress given immediately after training rats in the contextual fear conditioning task impaired subsequent retention levels (if given up to 3 hours after training, but not at 24 hours) (Rudy [101]; Rudy et al. [102]), but did not have any effect if applied to the auditory fear conditioning paradigm (Rudy [101]). However, auditory fear conditioning was facilitated by the administration of mild to medium intensity stressors (handling or subcutaneous vehicle injection) after training (Hui et al. [103]).

Retention levels for a particular type of classical conditioning paradigm, the conditioned taste aversion task (Garcia et al. [104]; Bermudez-Rattoni [105]), were also shown to be inhibited if a high stressor (forced swim) is given shortly after conditioning (Bourne et al. [106]).

*Conclusion*

The lack of homogeneity in the very few available studies for this category does not allow formulating any conclusions for the impact of posttraining extrinsic stress in Pavlovian conditioned memories.

### 4.3. Effects of acute extrinsic stress on the retrieval of information

#### 4.3.1. Spatial learning

A series of experiments has presented evidence for impairing effects of stress when it is given during a brief delay period between the acquisition of information and a subsequent retrieval challenge. Such delay normally lasts between 30 minutes and 4 hours, and therefore stress during such period can be influencing a variety of mechanisms, including consolidation, short-term memory, and retrieval. Using both conventional (Diamond et al. [107]) and water (Diamond et al. [108]; Woodson et al. [109]; Sandi et al. [110]) radial arm mazes, Diamond et al. have consistently shown that stress applied during such delay period interferes with subsequent retrieval of the previously acquired information. In most of their studies, the stressor applied was exposure of rats to a cat that, therefore, can be considered of high or very high intensity.

The same treatment was also effective to inhibit recall when it was given just immediately before the 24-hour memory test trial (Diamond et al. [98]). This finding fits with previous work in the Morris water maze, in which exposure to brief shocks 30 minutes, but not 2 minutes or 24 hours before testing (de Quervain et al. [111]). The same deleterious effect in retrieval of spatial information was observed by injecting corticosterone 30 minutes before retention testing (de Quervain et al. [111]). Further studies indicated that the impairing effects of glucocorticoids on retrieval of long-term spatial memory depend on noradrenergic mechanisms in the hippocampus, and moreover, that neuronal input from the BLA (and particularly norepinephrine-mediated BLA activity) is essential for the hippocampal glucocorticoid effects on memory retrieval to occur (Roozendaal et al. [112, 113]).

Convincing evidence indicates that the level of difficulty of the task (memory load) is a critical factor in observing the detrimental effects of stress on retrieval processes. Using the radial arm water maze, Diamond et al. [108] showed that exposure to a cat during a 30-minute delay period between training and testing for the platform location (the platform was located in the same arm on each trial within a day and was in a different arm across days) had no effect on memory recall in the easiest RAWM, but stress did impair memory in more difficult versions of the RAWM. By lesioning the hippocampus, the authors also confirmed that the radial arm water maze is a hippocampal-dependent task. In addition to the importance of memory load (difficulty or memory demand of the task), it seems that flexible forms of memory are particularly susceptible to show disrupted retrieval by stress, as opposed to more stable ones that remain largely unaffected (Célérier et al. [114]). This might reflect the differential susceptibility of different memory systems to be affected by stress.

Evidence for impairing effects of acute stress on subsequent/delayed retrieval has also been provided in humans, with emotionally arousing material being especially sensitive to this disruptive effect (Domes et al. [115]; Kuhlmann et al. [116]). As in animals, memory load is also an important factor for stress-induced retrieval impairments in humans (de Quervain et al. [117]).

*Conclusion*

The results reviewed here indicate that experiencing an acute, highly stressful, situation can interfere with information processing linked to retrieval of previously (recently) stored information. Although there is no information with regard to the impact of such extrinsic stress in tasks involving low intrinsic stress levels, we speculate that the inverted-U shape for the relationship between intrinsic stress and spatial information processing (Figure 3(b)) will be displaced to the left by the effect of extrinsic stress (see Figure 4(b)). Thus extrinsic stress would impair the retrieval of stressful spatial information (as described above), but would facilitate recall of spatial information linked to less arousing experiences. However, the left part of the curve remains speculative, and we cannot discard the other two possibilities of not finding an effect or even observing impaired spatial retrieval when extrinsic stress is applied before spatial tasks involving low intrinsic stress.

### 4.4. Neurobiological mechanisms involved in the acute effects of extrinsic stress on memory

The great sensitivity of the hippocampus to the disrupting effects of extrinsic stress in cognition is revealed by the profound suppression of hippocampal synaptic plasticity after acute exposure to stressors (Foy et al. [118]; Bennett et al. [119]; Diamond et al. [120]; Alfarez et al. [121]) or increased glucocorticoids (Alfarez et al. [121]). A crucial role for the medial temporal lobe (and the hippocampus in particular) in mediating these stress-induced retrieval impairments is also supported by human neuroimaging studies (de Quervain et al. [117]). In addition to the hippocampus, there is also evidence that acute stress-induced memory impairing effects can also be mediated by activation of dopaminergic (Murphy et al. [122]; Arnsten and Goldman-Rakic [123]) and noradrenergic (Birnbaum et al. [124]) transmissions in other structures known to be involved in high-order (including working memory and executive function) processing, such as the prefrontal cortex.

As to the potential molecular mechanisms, only a few studies have been reported. Reduced expression of NCAM in the hippocampus and prefrontal cortex after cat stress exposure was recently described to correlate with stress-induced retrieval deficits in the radial arm water maze (Sandi et al. [110]). These observations of a drastic reduction of NCAM in stressed memory-impaired rats is consistent with an increasing body of data indicating that NCAM is important for optimal circuit functioning and synaptic plasticity (Kiss et al. [125]; Welzl and Stork [126]; Washboume et al. [127]).

## 5. THE IMPACT OF CHRONIC EXTRINSIC STRESS ON MEMORY FUNCTION

Prolonged exposure to stress is recognized as a condition that can induce deleterious effects on brain structure and cognition (McEwen [128, 129]), as well as increasing the risk to develop neuropsychiatric disorders (Mazure [130]; de Kloet et al. [131]; Nemeroff et al. [132]).

Nowadays, the study of chronic stress is probably the most popular in the field of stress' interactions with cognitive function. In the vast majority (if not all) of studies dealing with chronic stress, it is extrinsic stress, experienced in a prolonged manner, that is studied, and therefore, most of the studies on chronic stress and memory fall into this definition. As previously, we should start by defining how the above-mentioned factors account for chronic extrinsic stress conditions.

- Stressor duration: in this subsection, we deal with *chronic* stress.
- Stressor intensity: the contribution of this factor to the impact of chronic stress has not being systematically studied. When possible, we will try to estimate the stressor intensity in the different chronic stress protocols under discussion, according to the range used above: *low*, *medium*, *high*, and *very high*.
- Stressor timing with regard to memory phase: although, in theory, one could imagine situations in which chronic stress is experienced at different times with regard to the different memory phases, virtually all studies in the literature applied stress procedures before exposing animals to any cognitive challenge. Therefore, we will group them in this review under the subheading of acquisition of information, even though all different memory phases could still be affected when stress is applied before learning.
- Learning type: we will deal with examples for both *Pavlovian conditioning* and *spatial learning*.

Summarizing, in this subsection, we will evaluate how chronic stress experienced before the learning challenge affects memory (both implicit and explicit types of memory) function.

### 5.1. Effects of chronic extrinsic stress on the acquisition of information

#### 5.1.1. Pavlovian conditioning

To our knowledge, the impact of chronic stress in Pavlovian conditioning in rodents has only been tested in fear conditioning protocols. Chronic restraint stress has been repeatedly shown to potentiate both contextual (Conrad et al. [133]; Sandi et al. [134]; Cordero et al. [135]) and auditory (Conrad et al. [133]) fear conditioning in rats. In all cited cases, the chronic stress procedure applied can be considered of high stress intensity (restraint stress: 6 h/day) and was applied during 21 consecutive days. Shorter exposure to chronic restraint stress (1 week) was ineffective to affect subsequent auditory fear conditioning; however, it impaired fear extinction applied 24 hours after conditioning (Miracle et al. [136]).

*Conclusion*

**Figure 5:**
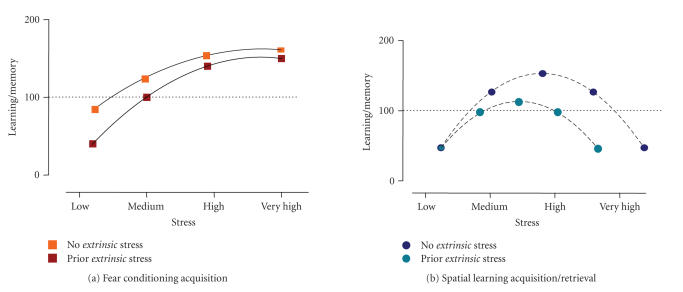
Impact of “chronic extrinsic” stress on memory formation. Chronic stress potentiates fear conditioning (a) and impairs spatial and reversal learning processes (b).

Chronic stress (high stressor intensity, 21-day duration) seems to facilitate fear conditioning processes (Figure 5(a)).

*Neurobiological mechanisms*

In the facilitating effect of fear conditioning induced by chronic stress, corticosterone has been proposed to play a mediating role (Conrad et al. [137]). At the neurobiological level, increasing evidence at the cellular and molecular levels suggests a connection between neuronal remodeling in the amygdala and the development of anxiety-like behavior (Vyas et al. [138, 139]; Mitra et al. [140]), which fits with the role of the amygdala in emotional behavior and fear (Phelps and LeDoux [141]). Restraint stress has been reported to enhance anxiety, and also to cause an increase in dendritic length and spine density in the BLA, but a reduction in the medial amygdala (Vyas et al. [138, 139]; Mitra et al. [140]). At the molecular level, recent evidence indicates that the serine protease tissue-plasminogen activator (tPA) (a key mediator of spine plasticity which is also required for stress-induced facilitation of anxiety-like behavior (Pawlak et al. [142])) plays a permissive role in the reported stress-induced spine loss in the medial amygdala (Bennur et al. [143]).

#### 5.1.2. Spatial learning

Since chronic stress was originally reported to damage hippocampal structure (McEwen [128, 129]), the possibility that chronic stress affects hippocampal-dependent learning has been extensively tested over the past years. Chronically stressed male rats were shown to exhibit learning and memory deficits in a variety of spatial tasks, including the radial-arm maze (Luine et al. [144]), the Y-maze (Conrad et al. [56]), and the Morris water maze (Venero et al. [145]; Sandi et al. [146]). Similarly, psychosocial stress consisting of rats' exposure to a cat for 5 weeks and randomly housed with a different group of cohorts each day was shown to exhibit impaired learning and memory in the radial-arm water maze (Park et al. [147]). Reversal learning in spatial tasks, a cognitive operation that in addition to the efficient use of spatial information requires flexibility to relearn a new platform, seems to be compromised following treatments involving chronic (21–28 days) glucocorticoid elevations (Sandi [4, 5]; Cerqueira et al. [148]).

There is no consensus as to whether periods of stress exposure shorter than the more or less standard protocol of 21 days would result in impaired learning. Luine et al. [149] reported that when restraint stress was given for 6 h/day for 7 days and spatial learning in the eight arm radial maze was evaluated on days 10–13 post stress, no effect on performance was noted; however, daily restraint stress for 13 days induced a medium enhancement of performance on days 10–13 post stress. More recently, Radecki et al. [150] showed that chronic immobilization stress (2 h/day × 7 days) in Long-Evans rats significantly impaired spatial performance in the Morris water maze, elevated plasma corticosterone, and attenuated hippocampal LTP.

*Conclusion*

Chronic stress (high stressor intensity, 3–5-week duration) seems to impair spatial and reversal learning.

*Neurobiological mechanisms*

Given that the hippocampus was originally found to be a main target of glucocorticoids and to be responsive to stress, much work on the neurobiological impact of stress has focused on this brain region. The idea behind is that, to certain extent, structural and molecular alterations (see below) induced by chronic stress in this brain area will account for the impairing effects of stress in hippocampus-dependent memory tasks (notably including spatial learning). Moreover, recent work is providing increasing evidence for parallel alterations induced by chronic stress in the prefrontal cortex, which could account also for some of the behavioral alterations described above and, specially, for stress-related impairments in reversal learning.

Briefly, cumulative work indicates that chronic stress markedly affects the hippocampal morphology. Stress and high glucocorticoid levels can suppress neurogenesis in the dentate gyrus (Gould and Tanapat [151]) and compromise cell survival (Sapolsky [152]). In the CA3 area, chronic stress has been shown to result in the following structural alterations: (i) dendritic atrophy of apical pyramidal neurons (Watanabe et al. [153]; Magariños and McEwen [154]); (ii) synaptic loss of excitatory glutamatergic synapses (Sousa et al. [155]; Sandi et al. [146]); (iii) a reorganization at the microstructural level within mossy fibre terminals (Magariños et al. [156]); (iv) a reduction in the surface area of postsynaptic densities (Sousa et al. [155]); and (v) a marked retraction of thorny excrescences (Stewart et al. [157]). In the CA1 area, the structural changes reported after chronic stress include (i) a general decrease of the dorsal anterior CA1 area's volume (Donohue et al. [158]); (ii) alterations in the lengths of the terminal dendritic segments of pyramidal cells in rat CA1 (Sousa et al. [155]); and (iii) an increase in the surface area of the postsynaptic density and volume in CA1 stratum lacunosum moleculare (Donohue et al. [158]).

Intriguingly, recent studies have suggested that spatial memory deficits may arise from HPA axis dysregulation following hippocampal damage, rather than being a direct effect of hippocampal injury. Thus, spatial memory deficits following CA3 hippocampal lesion could be prevented with a single injection of metyrapone, a corticosterone synthesis blocker, just before performance in the water maze (Roozendaal et al. [159]). Furthermore, the deleterious effects induced by a 21-day chronic restraint stress procedure in the Y-maze have been proposed to depend on corticosterone elevations at the time of behavioral assessment, since impaired performance was inhibited by pretesting metyrapone injections (Wright et al. [160]).

As to the prefrontal cortex, major neuronal remodeling occurs in its medial part as a consequence of chronic stress or prolonged glucocorticoid treatment, including dendritic atrophy (Wellman [161]; Cook and Wellman [162]; Radley et al. [163]; Liston et al. [164]) and spine loss (Cerqueira et al. [148]; Radley et al. [165]) in layers II/III.

Finally, given that the amygdala can exert important modulatory actions in hippocampus-dependent memory tasks (McGaugh [36]), further studies are needed to assess whether sensitization of amygdala activation induced by chronic stress (see above) might also participate in the reported spatial memory impairments.

At the molecular level, a large list of molecular mechanisms appears to contribute to the impairing actions of stress in brain structure and cognitive function. They include excitatory amino acids and a variety of signal transduction pathways, neurotrophic factors, and cell adhesion molecules (Sandi [4, 5]; McEwen [128]; Sapolsky [152]; Molteni et al. [166]).

## 6. DISCUSSION AND CONCLUSIONS

The results reviewed here emphasize the great importance of integrating different factors into a model of stress actions in memory formation. The five factors proposed and analyzed (see Section 2) seem to be critical to define the outcome of stress effects in memory processes.

The factor source of stress, distinguishing between *intrinsic* and *extrinsic* stress is the key to understand the complexity of effects and mechanisms involved. *Intrinsic* stress facilitates memory consolidation processes, whereas the effect of *extrinsic* stress in memory consolidation seems to be quite heterogeneous, and therefore, specifying the source of stress helps clarifying the claimed differential effects of stress/glucocorticoids in memory consolidation versus retrieval (Roozendaal [20]).

A second highly critical factor is the learning type under study, with high stress (both intrinsic and extrinsic) consistently facilitating *Pavlovian conditioning*, while high-to-very-high stress generally impairing the processing of *spatial information* (or relational and explicit types of learning). The latter proposal (i.e., that high-to-very-high stress impairs learning) is quite controversial since some researchers criticize the simplistic view that stress impairs learning by noting that the physiological stress response is a mechanism to optimize survival, and they propose that it is the behavioral strategy that changes under high stress conditions (de Kloet et al. [2]; Joëls et al. [8]). Although we basically agree with such interpretation, we should also recognize that when spatial learning/retrieval is under study, high-to-very-high stress conditions result in impaired performance in this type of tasks. It would be interesting to investigate whether such deleterious effect is in benefit of a facilitation of alternative learning (notably, emotional learning) types.

The factor “stressor intensity” is useful and allows making interexperiment comparisons. It also helps understanding how different magnitudes of challenge interact with cognition. Whereas the whole grading of stressor intensities is important to define the impact of intrinsic stress (see, e.g., Figure 3), it is high stress conditions which are particularly effective and representative of the impact of extrinsic stress in memory function.

The factor stressor timing with regard to memory phase is also critical, as we concluded that different memory phases show different vulnerabilities to stress. Although this was noted in many instances, a clear example is the susceptibility of Pavlovian conditioning to be facilitated when extrinsic stress is given before learning, but not afterwards (see Figure 4(a)), whereas it is the retrieval phase of spatial learning which seems to be particularly vulnerable to the impact of (acute) extrinsic stress.

Finally, the factor “stressor duration,” distinguishing between acute and chronic stress situations, although it give a similar outcome when observing its impact in memory function (cf. Figures 4 and 5), it makes a clear contribution when we talk about performance during “acquisition” of information. Whereas chronic extrinsic stress frequently has an impact on spatial learning, acute extrinsic stress normally does not affect spatial learning, but has been revealed to be more efficient to disturb retrieval.

Given the importance of other factors already mentioned throughout the review, such as the amount of effort/load included in the information processing (Diamond et al. [108]; Célérier et al. [114]), or individual differences in personality or other stress-relevant factors (Touyarot et al. [167]; Márquez et al. [29]), future integrative attempts should be directed to analyze and integrate these or other factors with the final goal of developing an integrative and reliable model that accounts for the whole complexity of stress interactions with cognition.

Summarizing on those conditions in which we have enough information to compare the integrated impact of the different factors analyzed, we could conclude that high stress levels, whether intrinsic or extrinsic, tend to facilitate Pavlovian conditioning (in a linear-asymptotic manner), while being deleterious for spatial/explicit information processing (which with regard to intrinsic stress levels follows an inverted U-shape effect). We consider this integrative model more explanatory than classifications performed among individual factors (see Section 1).

As to the neurobiological mechanisms, a common observed feature seems to be a key role of glucocorticoids in mediating both the facilitating and impairing actions of stress in different memory processes and phases. Among the brain regions implicated, the hippocampus, amygdale, and prefrontal cortex were highlighted as critical for the mediation of stress effects. Further work is needed to develop a mechanistic explanatory model at the neurobiological level that accounts for the different interactions and factors discussed above.
